# An integration of phenotypic and transcriptomic data analysis reveals yield-related hub genes in *Jatropha curcas* inflorescence

**DOI:** 10.1371/journal.pone.0203441

**Published:** 2018-09-21

**Authors:** Nisha Govender, Siju Senan, Edison Eukun Sage, Zeti-Azura Mohamed-Hussein, Mukram Mohamed Mackeen, Ratnam Wickneswari

**Affiliations:** 1 School of Environmental and Natural Resource Sciences, Faculty of Science and Technology, Universiti Kebangsaan Malaysia, UKM Bangi, Selangor, Malaysia; 2 Institute of Systems Biology (INBIOSIS), Universiti Kebangsaan Malaysia, UKM Bangi, Selangor, Malaysia; 3 School of Chemical Sciences and Food Technology, Faculty of Science and Technology, Universiti Kebangsaan Malaysia, UKM Bangi, Selangor, Malaysia; 4 School of Biosciences and Biotechnology, Faculty of Science and Technology, Universiti Kebangsaan Malaysia, UKM Bangi, Selangor, Malaysia; Institute of Genetics and Developmental Biology Chinese Academy of Sciences, CHINA

## Abstract

*Jatropha curcas* is an oil-rich seed crop with huge potentials for bioenergy production. The inflorescence carries a number of processes that are likely to affect the overall yield potentials; floral development, male-to-female flower ratio, floral abscission and fruit set. In this study, a weighted gene co-expression network analysis which integrates the transcriptome, physical and simple sugar data of *J*. *curcas* inflorescence was performed and nine modules were identified by means of hierarchical clustering. Among them, four modules (green4, antiquewhite2, brown2 and lightskyblue4) showed significant correlation to yield factors at p≤0.01. The four modules are categorized into two clusters; cluster 1 of green4 and antiquewhite2 modules correspond to number of flowers/inflorescence, total seed weight/plant, number of seeds/plant, and number of fruits/plant, whereas cluster 2 of brown2 and lightskyblue4 modules correspond to glucose and fructose. Descriptive characterizations of cluster 1 show putative involvement in gibberellin signaling and responses, whereas cluster 2 may have been involved in sugar signaling, signal transductions and regulation of flowerings. Our findings present a list of hub genes for *J*. *curcas* yield improvement and reproductive biology enhancement strategies.

## Introduction

*Jatropha curcas* L. is a monoecious shrub with huge potentials for biofuel production [1, 2]. A global increase in human population and living standards is expected to raise the future energy demands. Derived from transesterification, the EN 12214 grade Jatropha methyl ester (biodiesel) has ideal iodine and saponification values [3] and therefore, it has been marked as an essential renewable alternative for energy security. At present, the success rate of Jatropha-based biodiesel is relatively poor due to low and inconsistent crop yield affected by unreliable flowering and low seed production [4, 5]. An extensive amount of genetic and molecular data has suggested that by manipulating the fruit, seed, flower and other plant reproductive organs, the yield and subsequent harvesting index could be enhanced tremendously [6,7, 8].

In *J*. *curcas*, the inflorescence has a profound impact on later stages of plant reproductive biology, and not much is known about the genes regulating these biological processes. A typical *J*. *curcas* has an average male-to-female ratio of 29:1 [9] with staminate and pistillate flowers borne on the same inflorescence [10]. Flowering and seed setting improvements would significantly affect the plant seed yield and hence the oil yield: greater number of female flowers, a lower rate of female floral abscission and efficient fruit setting qualities. Several female flowering-related and seed-related genes in *J*. *curcas* have been described. As such, the JcLFFY gene was shown to regulate flower identity, floral organ patterns and fruit shape [11] whereas the CYP78A98, a cytochrome P450 gene demonstrated a control over the Jatropha seed and fruit size [12]. Dissecting complex traits such as yield is particularly challenging because these traits are underpinned by a group of genes, also named the hub genes/modules. The inter-connected genes in a module work together to co-ordinate and determine the phenotypic endpoints. With a co-expression gene network, revealing the putative relationships between genes that control a given trait is likely to contribute to a comprehensive understanding of complex biological processes such as the *J*. *curcas* yield potentials.

Photosynthesis by green plants fixes carbon dioxide into sugars. Subsequently, the assimilated carbon is transported to sink tissues to support plant physiological processes during growth and development; seed germination, flowering initiation, flowering, fruit and seed set, fruit maturity, senescence and protective functions against various abiotic stresses [13, 14]. The heterotrophic sink organs utilize sugars for growth and/or otherwise stores the carbon compound. Sugars are dual-role primary metabolites (nutrients and regulators) that serve as precursors for anabolic reactions, intercellular signaling mechanisms, structural polymers and complex systems such as the carbon partitioning regulation, carbohydrate and lipid metabolism, gene expression, protein synthesis and osmotic homeostasis [14, 15, 16]. In plants, sucrose, glucose and fructose are simple soluble sugars that work collectively to maintain the overall structure and growth of plants [17]; glucose and fructose are involved in cell division whereas sucrose promotes differentiation and maturation [18, 19]. The different concentrations of soluble sugars in plant tissues were shown to affect embryogenesis [16], organ formation and functions in inflorescence, leaf, and tuber [20] and proliferation of organs (increase in size, number and/or thickness of plant parts) [16, 21].

Metabolic requirements in different plant tissues at different developmental stages change dynamically with time and according to environmental conditions. Sugars are the primary energy source for the formation of reproductive structures [22, 23] and therefore the success of reproductive growth is predominantly dependent on the concentration of simple soluble sugars. Molecular technologies are rapidly developed and widely applied in the search for genes that influence a given trait. Alongside, systems genetics approach is used for identification of genes/pathways underlying complex traits such as yield factors. In this work, we first constructed RNA-sequencing (RNA-seq)-based libraries for *J*. *curcas* inflorescence and the phenotypic data which includes the physical parameters and simple soluble sugar concentrations of the plants/inflorescences were recorded. The weighted gene co-expression network analysis (WGCNA) [24, 25] was employed for the identification of modules corresponding to *J*. *curcas* yield factors. Here, a comprehensive description of hub genes responsible for yield factors in *J*. *curcas* inflorescence is given. These findings impart an important knowledge-base for *J*. *curcas* yield enhancement breeding approaches.

## Materials and methods

### Plant materials

A total of six individual J. curcas plants with parallel flowering time were selected at Experimental Plot A, Universiti Kebangsaan Malaysia (UKM), Bangi (2°5509.0N101°4704.8E). Each two-year-old plant belongs to the following accessions: two plants from UKM JC-17 accession and UKM JC-18 accession, respectively and a single plant from each of the UKM JC-20 and UKM JC-21 accessions. All plants utilized in this study were watered and fertilized regularly. During the collection (June 2014), plants were healthy and any visible external symptoms associated with biotic and abiotic factors were completely absent. The *J*. *curcas* inflorescence was detached at the base of the peduncle using a sharp knife. The fresh cut inflorescence was washed briefly, cut into small pieces prior to RNA-seq library preparation and simple soluble sugar analysis. Each plant and their corresponding inflorescence were evaluated for the number of flowers per inflorescence, number of fruits per plant, number of seeds per plant and total seed weight (g) per plant.

### Quantification of simple soluble sugars

Simple soluble sugars in *J*. *curcas* inflorescence were extracted according to Dickson 1979 [26] with several modifications for quantitative analysis. An inflorescence with approximately 10 g fresh weight was ground into paste using a Blender (Kenwood). The dissolved paste (in 50 ml of MiliQ water) was subjected to incubation for 8 h at 80°C with shaking (150 rpm). The solution was filtered using a Whatmann paper. The filtrate was collected and 5 ml of n-hexane was added. Incubation was repeated at 40°C for 8 h with shaking (150 rpm). The solution was allowed to stand still for 30 min. When two distinct layers were observed, the bottom aqueous layer was collected and dried at 30°C for an overnight. The residue (about 0.2–0.5 g) was suspended into 2 ml of 1:1 acetonitrile (ACN): water. The solution filtered using a syringe and nitrocellulose membrane filter (0.22 μm) was stored in a vial. Samples were run on an HPLC (Agilent 1100 series) instrument coupled to an evaporative light scattering detector (ELSD) (Alltech ELSD 2000) using the Shodex HILICpak VG-50 4E column. The sample injection volume was 10 μl and an isocratic run time was set for 20 min using ACN: water (1:4) as the mobile phase. The ELSD detector parameters were as follows: drift tube temperature was fixed at 80°C, nitrogen gas flow was set at 1.8 slm (standard liter per minute), detector gain was fixed at 1, and the impactor mode was turned off. The commercial simple soluble sugars were prepared as standards (serial concentrations) for the quantification of glucose, fructose and sucrose in each sample based on the HPLC peak area. The limit of detection (LOD) and limit of quantitation (LOQ) values for each standard were computed according to ICH Harmonised Tripartite Guideline 2005 [27].

### RNA isolation, sequencing and assembly

Total RNA was isolated from each sample using a combined modified method; CTAB+silica column [28] and RNeasy Plant mini kit (Qiagen, Hilden, Germany). The total RNA concentration was measured by Nanodrop ND-10000 spectrophotometer (NanoDrop Technologies, USA). The quality and integrity of the total RNA were determined at 260/280 nm ratio by NanoBioanalyzerRNA-6000 analysis (Agilent-Technologies). The cDNA library was constructed according to the SureSelect Strand-Specific RNA Library Preparation for Next Generation Sequencing workflow protocol (Agilent Technologies, USA) and the corresponding fragmented genomic DNA was sequenced by Illumina HiSeq 2500 (Yourgene Bioscience, Taiwan). The output, raw fastq reads were trimmed using the Trim Galore package and aligned to *J*. *curcas* genomic sequences obtained from Kazusa's Jatropha Genome Database version r4.5 (ftp://ftp.kazusa.or.jp/pub/jatropha/). Alignment was performed using the STAR aligner (https://github.com/alexdobin/STAR) and the Cufflinks was used to produce a merged assembly. Assembly construction was performed using the CLC Genomics Workbench (CLC Bio, USA). For descriptive characterization, the *J*. *curcas* reads were annotated using BLAST2GO PRO 4.0 suite [29, 30]; BLAST, mapping, InterPro Scan and annotation.

### Gene co-expression network construction

Normalization of count data into counts per million (cpm) was performed using the edgeR [31, 32] and limma [33] packages (R library). Lowly expressed genes, set at a cpm value <5 in all samples were filtered out. Six inflorescence transcriptomes were clustered using the average method to detect presence/absence of outliers among the biological replicates. A gene co-expression network was constructed using the weighted gene co-expression network analysis (WGCNA) package [24, 25] as follows; i) perform pair-wise comparisons of gene expression values across all samples by calculating the Pearson correlation coefficient ii) raise the correlation matrix to a power (β) function for transformation into a mathematically represented adjacency matrix iii) identify modules (group of genes with high Topological Overlap) by means of hierarchical clustering (each module is indicated by a colour) iv) relate modules to traits by testing the association between the module eigengene (ME) and the trait of interest v) examine the intra-modular activity of candidate modules using the hierarchical clustering. A co-expression network is comprised of nodes that represent genes, and the edges between the nodes represent the interactions between the pairs of genes. The module networks were drawn, visualized and annotated using the Cytoscape 3.0 software, an open source java script package and the corresponding topological measures were calculated by Network analyzer [34].

#### Characterization of functional modules

Gene members of each module were characterized according to the KaPPA-View4 Jatropha (http://kpv.kazusa.or.jp/kpv4) metabolic pathway database [35] and literature. The clusters of modules were comparatively categorized in accordance to the specific sub-cellular compartments (cellular component, molecular function and biological process) as defined by Gene Ontology (GO) using the Web Gene Ontology Annotation Plot (WEGO) [36].

## Results and discussion

### *Jatropha curcas* inflorescence and the corresponding yield factors

**Fig 1:**
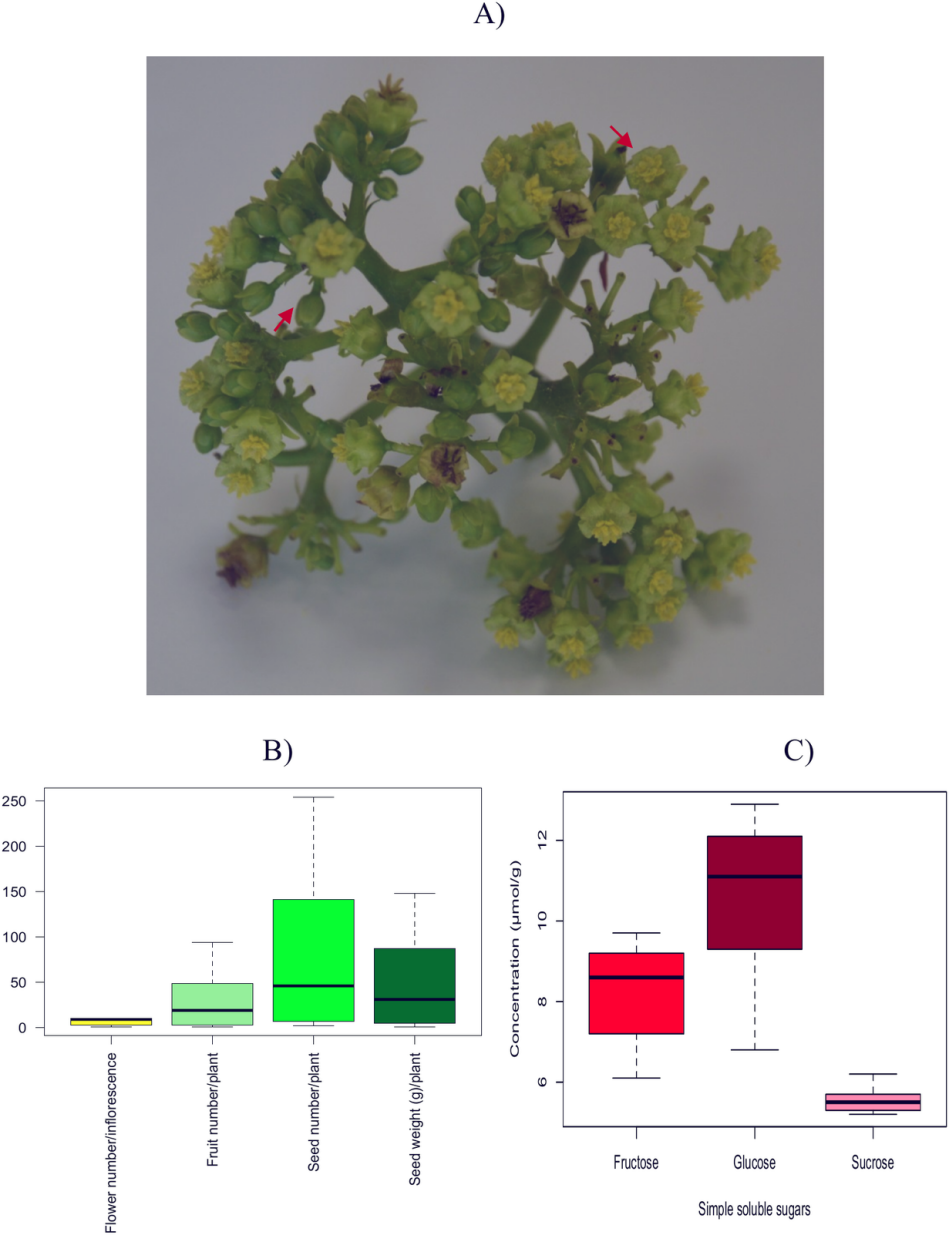
The *Jatropha curcas* inflorescence: Physical data and simple soluble sugars. A) A two-year-old mature inflorescence obtained from the UKM JC-17 accession. Red arrows indicate buds and bloomed flowers. B) Box plots indicate metric quantifications of flower number per inflorescence, fruit number per plant, seed number per plant and seed weight expressed in grams per plant. C) Box plots indicate concentrations of fructose, glucose and sucrose in *J*. *curcas* inflorescence expressed in μmol/g. All measurements (B and C) for each treatment were taken from 5 biological replicates.

*Jatropha curcas* inflorescence is the site where reproductive processes take place and that each process is likely to affect the plants yield potential:flowering, floral organ development and sex differentiation, pollination, pollen tube entry, growth and development, and fruit set and maturation. There are three types of inflorescence classified according to their male to female flower ratio; male, female and middle. The male type is characterized with no female flowers, whereas both the middle and female types have a male: female flower ratio of 94:26 and 79:8, respectively [37]. Accordingly, the male and middle type inflorescence comprised of buds, anthesed flowers and dehisced flowers arranged in a dichasial or polychasial cyme pattern (Fig 1A) were used for both transcriptome and simple soluble sugar analyses because all plants in our experimental field were producing either a male or a middle type inflorescence only. Based on our phenology observation, an inflorescence with a combination of buds, anthesed and dehisced flowers represented the late maturity stage. Following the late maturity stage of the inflorescence, the fruit initiation and subsequent fruit and seed setting stages were observed (data not shown). The physical traits (yield factors) for each *J*. *curcas* plant and inflorescence, were obtained as following: the maximum (max) and minimum (min) number of flowers per inflorescence (FPI) was 10 and 1, respectively and the average number of FPI was 6. The number of fruits per plant (FPP) ranged in between 1 to 94, and with an average FPP of 33 (Fig 1B). The FPP values obtained in this study was comparable to others found in different geographic region: 15-month old *J*. *curcas* grown in Vadodara, India produced an average FPP of 20 [10] whereas in Senegal, 42-month old plants showed an average FPP of 31 (dry season) and 394 (wet season) [38]. Seed number per plant (SPP) showed a broad range at 2 (min) and 254 (max) and their corresponding seed weight per plant (SwPP) ranged at 1 (min) to 148 (max) g. The average values of both SPP and SwPP were 90 and 54 g, respectively (Fig 1B). Overall, the physical data obtained from our *J*. *curcas* plants were comparable to previously reported findings from Malaysia [39], Senegal [38], South Africa [40] and India [41].

### Simple soluble sugars in *J*. *curcas* inflorescence

Soluble sugars affect the plant structure and metabolism at the cellular, tissue, system and organism levels. They act as nutrient and metabolite signaling molecules for modifications of gene expression and proteomic patterns [18, 19, 42] and are directly linked to central metabolic reactions in plants such as mitochondrial respiration, photosynthesis regulation, reactive oxygen species balance, transport and heterotrophic utilization [43, 44]. The standard curve for each simple sugar and their corresponding limit of detection (LOD) and limit of quantitation (LOQ) are presented in S1 Fig. Concentrations of simple soluble sugars (monosaccharides: glucose, fructose and disaccharide: sucrose) in *J*. *curcas* inflorescence ranged from 5 to 13 μmol/g. Among the three sugars, the concentration range of glucose was highest at 6.8–12.9 μmol/g (average value = 10.4 μmol/g). This was followed by the concentration range of fructose at 6.1–9.2 μmol/g (mean value: 8.2 μmol/g). Sucrose showed the lowest concentration range among the simple soluble sugars at 5.2–6.2 μmol/g (average value = 5.6 μmol/g) (Fig 1C). The concentration of sucrose, also the plant systemic transport sugar, was the lowest in comparison to its counterpart monomers (glucose and fructose) suggests possible cleavage of pre-existing sucrose molecules. The cleavage of a sucrose molecule produces fructose and glucose and in the case of glucose, subsequent activation may form UDP-glucose. A low concentration of sucrose in *J*. *curcas* inflorescence suggests probable production of signal molecules such as the glucose, UDP-glucose and fructose [45, 46]. These sugar signals may have played a role in regulating growth and floral transition [47] in *J*. *curcas* inflorescence. In Arabidopsis, the specifically-cleaved sucrose molecules was shown to affect the inflorescence architecture and flowering time [48]. In the same species, sucrose has also demonstrated an imperative role in regulating the meristem identity genes such as LEAFY [21], cell division [49], meristem maintenance of shoots [50], roots and shoot meristem size [48, 51]. In this study, the simple soluble sugars quantified at a relative ratio of 1.8:1.5:1 (*glc*: *fru*: *suc*) in *J*. *curcas* inflorescence were applied for a gene co-expression network analysis to identify candidate genes corresponding to sugar modules.

### Transcriptome analysis of *J*. *curcas* inflorescence

**Fig 2:**
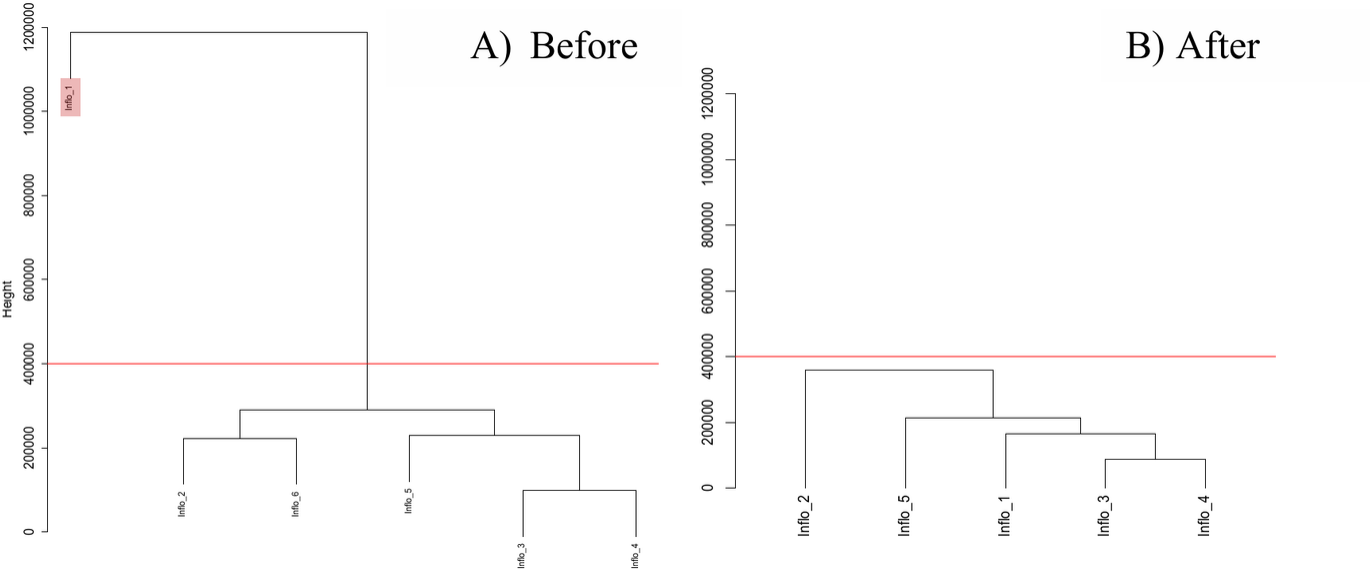
Detection for *Jatropha curcas* (inflorescence) outlier samples. A) Cluster of dendrogram indicates presence of an outlier (red box) above the cut-off height = 400 000. B) After the removal of an outlier, the dendrogram of cluster indicates homogeneity among the samples below the cut-off height.

The transcriptome analysis revealed a total of 62 889 reads from the six libraries generated from *J*. *curcas* inflorescence and the average assembly size ranged from 16 886 160–26 833 093 reads. The reads were subjected to BLAST2GO PRO 4.0 suite [29, 30] for descriptive characterization; BLAST, mapping, InterPro Scan and annotation. From the 21 188 sequences with BLASTX hits, only 4783 and 30 113 reads acquired mapping and annotation, respectively. The InterPro Scan (IPS) revealed 34 438 sequences without an IPS match, while 29 741 and 16 543 sequences scored with IPS and with GOs, respectively. (Table 1). The raw data of *J*. *curcas* inflorescence transcriptomes are found in Sequence Read Archive (SRA), National Center for Biotechnology (NCBI) database with the accession number SRP090662 (https://www.ncbi.nlm.nih.gov/sra/?term=SRP090662). Lowly expressed genes were filtered from the RNA-seq count data set and 12, 171 genes were fed into subsequent analysis. A cluster of dendrogram constructed with six inflorescence samples indicated presence of an outlier at height = 2000 (Fig 2). The outlier sample was removed and only five transcriptomes were applied for the weighted gene co-expression network construction.

**Table 1:** Descriptive statistics of the *Jatropha curcas* inflorescence transcriptomes.

Attributes	
Number of reads	62 889
Reads with BLASTX hits	21 888
Reads with mapping	4 783
Reads with GO annotation	30 113
Reads with IPS	29 741
Reads with IPS and GO terms	16 543

### Weighted co-expression gene network analysis (WGCNA) identifies novel modules

**Fig 3:**
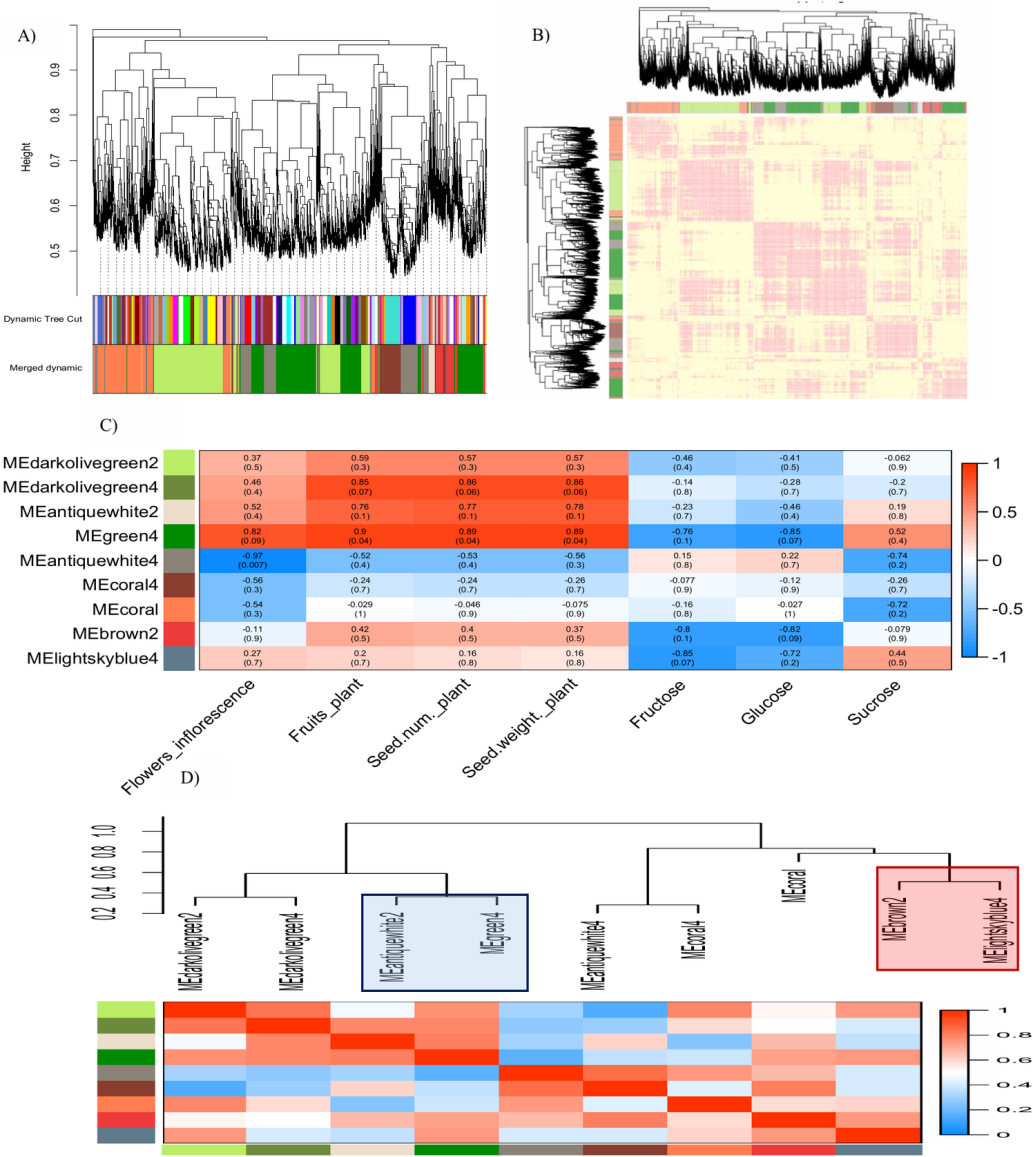
Weighted gene co-expression network analysis of RNA-seq based expression profiles of two-year-old *Jatropha curcas* inflorescences. A) Cluster of dendrogram indicates total number of modules under the dynamic tree cut and merged dynamic (pooled modules). B) Weighted gene co-expression network of the *J*. *curcas* inflorescence. C) Correlations between the module eigengenes and yield factors (traits); flowers per inflorescence, fruits per plant, seeds per plant, total seed weight (g) and simple soluble sugars (fructose, glucose and sucrose). Column represent the yield factors and the row represent module eigengenes (MEs). Correlation coefficient is represented by colours ranging from red (high positive correlation) to green (high negative correlation) and the corresponding p-values are presented in parentheses. D) Correlations between the MEs represented by dendrogram (top) and heatmap (bottom). Blue box and red box in the dendrogram represents cluster 1 and 2, respectively. Column and row in the heatmap represent the module colour/module identity. Correlation coefficient is represented by colours as described in C).

The transcriptome data of the *Jatropha curcas* inflorescence produced a weighted gene co-expression network by means of hierarchical clustering in accordance to the following conditions and criteria: A soft thresholding power (β) was selected to construct the weighted gene network based on the approximate scale-free topology criterion. From a set of candidate powers (1–20), β = 12 returned a scale-free topology fit index of -0.6 (red line) and the R^2^ reached the plateau for the first time in comparison to other values (S2 Fig). An adjacency matrix, obtained at β = 12 was transformed into Topological Overlap Matrix (TOM). Our dendrogram identified 132 modules (dynamicColors) in *J*. *curcas* inflorescence with 30–439 number of genes in each module. Large modules with 432 to 439 numbers of genes were green and turquoise, whereas the small modules (dark olivegreen1, firebrick2, indianred2, lightblue3, lightskyblue, mediumpurple, mistyrose, orangered, pink3, sienna2, slateblue, yellow3) contained 30–39 genes. Based on the hierarchical clustering, modules with short distances were pooled and therefore, a merged version of nine modules (Merge-cut) was obtained; darkolivegreen2, darkolivegreen4, green4, antiquewhite2, antiquewhite4, coral4, coral, brown2, and lightskyblue4 (Fig 3A). A gene co-expression network model which follows a scale-free and power-law distribution was built (Fig 3B) and the Merge-cut modules were correlated to yield factors: concentrations of simple soluble sugars (glucose, fructose and sucrose), number flowers per inflorescence, number of fruits per plant, number of seeds per plant and total seed weight per plant. We used the Pearsons correlation coefficient (*r*) to measure the strength of linear relationships between the module eigengenes (MEs) and yield factors. The relationship between the MEs and yield factors were described using the correlation (strength) guide suggested by Evans (1996) [52] as following: 0<r≤0.19, very weak; 0.2<*r*≤0.39, weak; 0.4<*r*≤0.59, moderate; 0.6<*r*≤0.79, strong; 0.8<*r*≤1.00, very strong. A positive and negative value of *r* denotes positive and negative linear correlations between the two variables, respectively. We choose modules with strength higher than moderate (*r*≥0.4; between the traits and ME) at p≤0.1 for further characterization while others were omitted.

The ME represents the first principal component of a module and therefore, it corresponds to the overall expression of a module. In this study, the following modules were discarded for further analysis because the p values of their corresponding ME-trait (yield factor) correlations did not fulfill the cutoff values: darkolivegreen2, darkolivegreen4, antiquewhite2, coral4, and coral. Among the 9 modules generated from the weighted gene co-expression network of *J*. *curcas*, the green4 module showed the most appealing association to the following yield factors: number of flowers per inflorescence (hereafter, flowers/inflorescence), number of fruits per plant (hereafter, fruits/plant), seed number per plant (hereafter, seed number) total seed weight (g) per plant (hereafter, seed weight) and simple soluble sugars (fructose and glucose only). The correlation between the ME of green4 module and flowers/inflorescence (*r* = 0.82), fruits/plant (*r* = 0.9), seed number (*r* = 0.89) and seed weight (0.89) are very strong at p≤0.09 (Fig 3C). In contrast, the correlation between the ME of green4 module and fructose and glucose are negatively strong (*r* = -0.76, at p≤0.1) and very strong (*r* = -0.85 at p≤0.07), respectively. Besides the green4 module, both the MEs of brown2 and lightskyblue4 modules show a relatively strong to very strong correlation between fructose and glucose; the *r*-value between the brown2 ME and fructose is -0.8 (at p≤0.1) and the *r* value between brown2 ME and glucose is -0.82 (at p≤0.09), whereas the r values between the lightskyblue4 ME and fructose and glucose are -0.85 (at p≤0.07) and -0.72 (at p≤0.2), respectively. The antiquewhite4 module corresponds very strongly (*r* = -0.97 at p≤0.007) to the number of flowers/inflorescence (Fig 3C).

The inter-modular analysis differentiated the modules into two groups with four and five modules in each group. The first group comprise of the darkolivegreen2, darkolivegreen4, antiquewhite2 and green4 modules while the other contains antiquewhite4, coral4, coral, brown2 and lightskyblue4 modules. Modules characterized with satisfactory strength for ME-trait associations are found as interconnected modules; green4 and antiquewhite2 modules in cluster 1 and brown2 and lightskyblue4 modules in cluster 2. The interconnected clusters correspond to the following traits of interest: cluster1 for flowers/inflorescence, fruits/plant, seed number and seed weight whereas cluster2 for fructose and glucose. Strong correlation between the antiquewhite2 module and green4 module at *r* = 0.63 at p<0.01 suggest flowering and fruiting are two closely related, energy demanding processes. Likewise, strong correlation at *r* = 0.87 (p <0.01) between the brown2 module and lightskyblue4 module suggest that these modules collectively affects fructose and glucose concentration in *J*. *curcas* inflorescence (Fig 3D).

**Fig 4:**
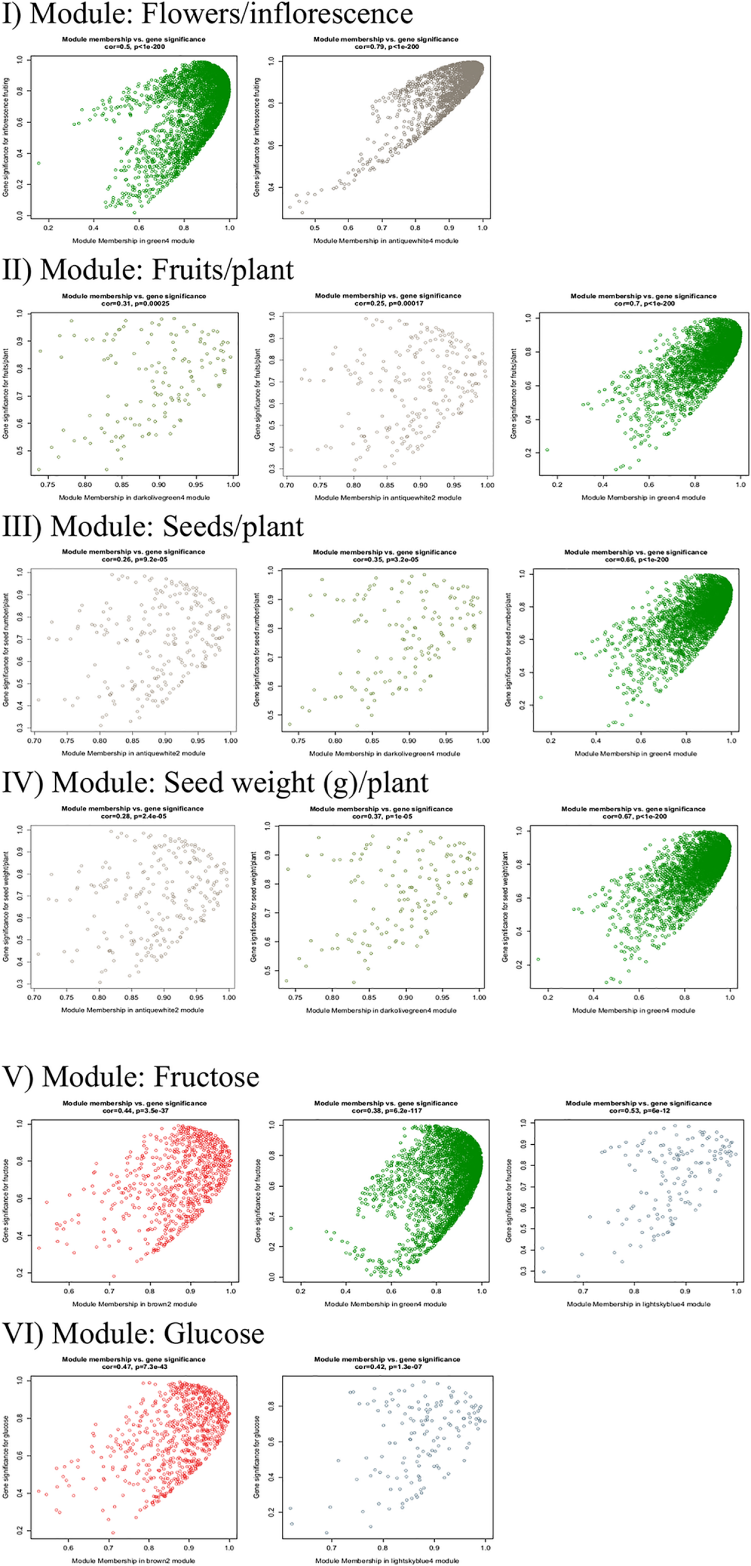
Gene significance (GS)-module membership (MM) of *Jatropha curcas*. Modules are obtained from hierarchical clustering of transcriptome data (inflorescence) and the GS-MM correlation (cor) is calculated using the weighted gene co-expression network analysis (WGCNA). Each trait and the putatively corresponding modules (in parentheses) are listed as following: I), flowers per inflorescence (antiquewhite4 and green4); II), fruits per plant (antiquewhite2, darkolivegreen4 and green4); III), seeds per plant (antiquewhite2, darkolivegreen4 and green4); IV), seed weight per plant (antiquewhite2, darkolivegreen4 and green4); V), fructose (brown2, green4 and lightskyblue4) and VI), glucose (brown2 and lightskyblue4).

For each module, we plotted the Gene Significance (GS) against the module membership (MM), defined as the correlation between the gene members (x_i_) within the module and the ME: MM(_i_) = cor (x_i_, ME) in a scatter plot. The MM measures the strength of a gene within the module, and therefore, the greater is the absolute value of MM, the stronger is the participation/influence of the gene within the module. For physical data, four corresponding modules (antiquewhite2, antiquewhite4, darkolivegreen4 and green4) obtained from the module eigen gene-trait correlation analysis were evaluated to determine their module membership (MM)-gene significance (GS) strength. Two modules namely the green4 (*r* = 0.5) and antiquewhite4 (*r* = 0.79), significantly corresponded to fruits/inflorescence at p<0.001. The MM of antiquewhite2, darkolivegreen4 and green4 modules were found correlated (moderately strongly to moderately weak) to the GS values of fruits/plant, seeds number and seed weight. Here, we only selected modules with a cutoff *r*>0.4 at p <0.001 for further characterizations. The MM of antiquewhite2 module showed weak correlation for GS values of fruits/plant (*r* = 0.31), seeds number (0.26) and seed weight (*r* = 0.28) at p <0.001. Likewise, the MM of darkolivegreen module showed weak correlation for GS values of fruits/plant (*r* = 0.25), seed number (*r* = 0.35) and seed weight (*r* = 0.37) at p <0.001. At *r*>0.6 and p <0.001, the MM of green4 module positively corresponded to GS values of fruits/plant, seed number and seed weight (Fig 4).

Our findings suggest that the flowers/inflorescence trait is underpinned by both green4 and antiquewhite4 modules whereas others such as the fruits/plant, seeds number and seed weight are collectively controlled by the green4 module only. Simple soluble sugar (glucose and fructose) are under the control of brown2 and lightskyblue4 modules. The MM of lightskyblue4 modules shows a moderately strong correlation (*r* = 0.53) between fructose GS and a moderately weak correlation (*r* = 0.42) to glucose GS. Likewise, the MM of brown2 module shows moderately weak correlation between the fructose and glucose GS values at *r* = 0.44 and r = 0.47, respectively. The MM of green4 module shows a moderately weak correlation (*r* = 0.38) between the fructose GS value (Fig 4).

Green4 module corresponds to the following traits: flowers/inflorescence, fruits/plant, seeds number and seed weight and fructose. The ME of green4 module showed positive correlation to physical traits and negative correlation to fructose. In addition, the green4 module indicated an interesting association between physical traits and fructose concentration in *J*. *curcas* inflorescence, whereby physical parameters operated in antagonist of fructose concentration. On the hand, antiquewhite4 module corresponding to flowers/ inflorescence was found not affecting any other traits significantly (Fig 4). Therefore, the molecular processes under this module may have been regulating floral morphogenesis processes, specifically.

### Gene Ontology (GO) classification of modules of interest

**Fig 5:**
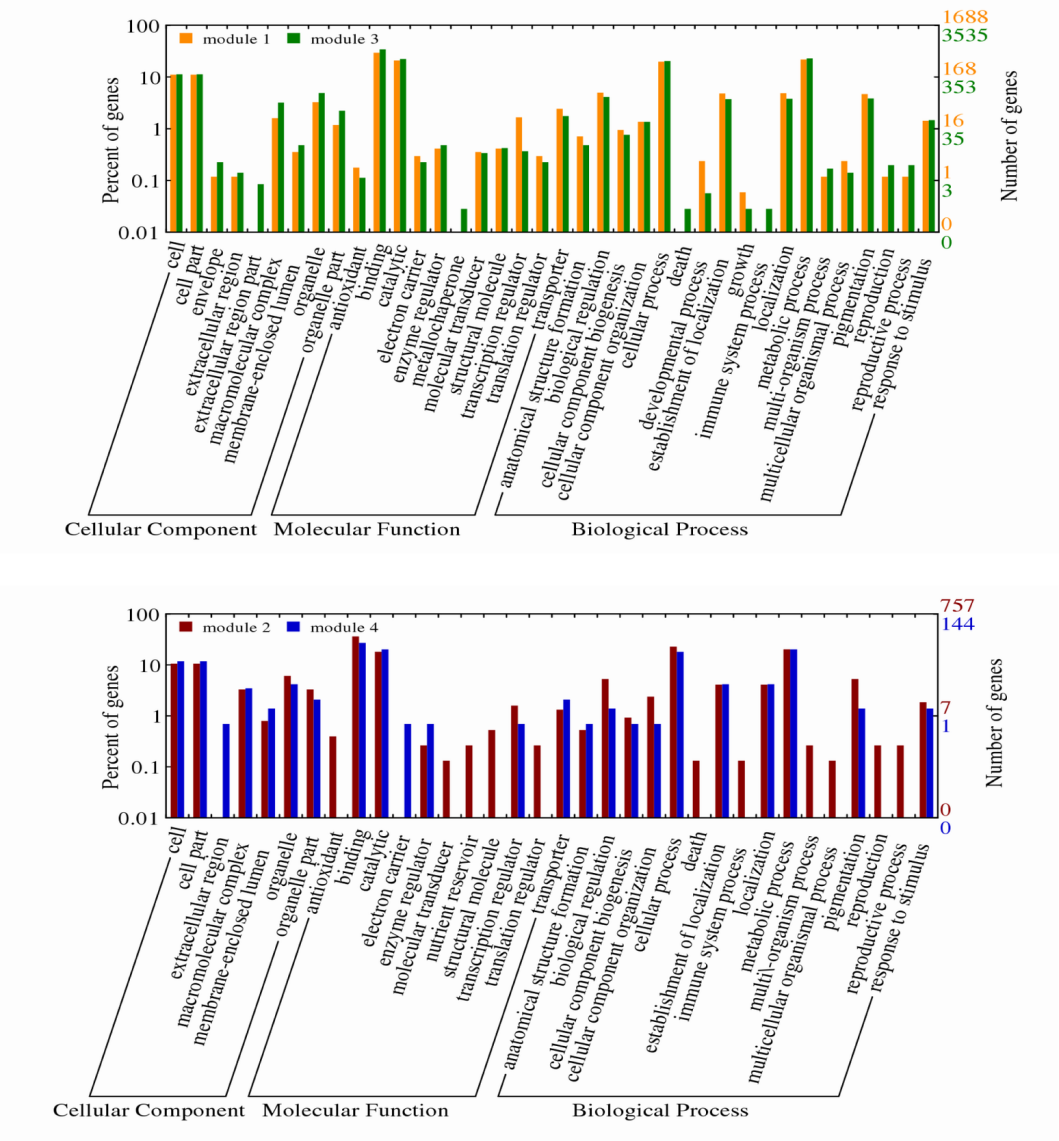
Gene Ontology (GO) term classification by cellular component, molecular function and biological process for the cluster 1 and cluster 2 modules obtained from weighted gene co-expression network analysis of *Jatropha curcas* inflorescence. Cluster 1 (top) contains module 1(antiquewhite2) and module 3 (green4) and cluster 2 (bottom) is comprised of module 2 (brown2) and module 4 (lightskyblue4).

Cluster 1 is comprised of module 1 (antiquewhite2) and module 3 (green4). The first module corresponds to flowers/inflorescence while the latter corresponds to fruits/plant, seed number and seed weight. Cluster 2 containing module 2 (brown2) and module 4 (lightskyblue4) corresponds to fructose and glucose. The Gene Ontology (GO) classification of cluster 1 grouped 1688 genes from the antiquewhite2 module and 3535 genes from the green4 module into cellular component (CC), molecular function (MF) and biological process (BP). In CC, both modules displayed highest (>10% of total genes) involvement in cell and cell part, followed by organelle, organelle part and macromolecular complex while under the MF, both binding and catalytic genes were highly expressed (>50% of the total genes). In biological processes, the highest involvement (with more than 40% of total genes of the modules) is found in cellular and metabolic processes. Other interesting biological processes found related to fruits/inflorescence includes the establishment of localization, multicellular organismal process, anatomical structure formation, biological regulation, cellular component biogenesis, cellular process, pigmentation, reproduction and reproductive process. The green4 module is distinguishable from the antiquehite2 module in respect to macromolecular complex, metallochaperone, death and immune system processes (Fig 5A).

Likewise, cluster 2 grouped 757 genes from the brown2 module and 144 genes from the lightskyblue4 module into various GO terms. The BPs of brown2 module and lightskyblue4 module were similar for the establishment of localization, localization and metabolic process. The most common GO terms observed among the brown2 module and lightskyblue4 module were cell and cell part (at >10% of the total genes of the modules) from CC, binding and catalytic (at 60% of the total genes of the modules) from MF and cellular and metabolic processes (at 40% of the total genes of the modules) from BP. The brown2 module is distinguishable from the lightskyblue4 module in MFs (antioxidant, molecule transducer, nutrient reservoir, structural molecule and translation regulator) and BPs (death, immune system process, multi-organism process, multicellular organism process, reproduction and reproductive process). In contrary, the lightskyblue4 module is distinctive from module 2 in extracellular region (CC) and electron carrier (MF) only. Binding and catalytic activities are highly common in both clusters (Fig 5B).

#### Characterization of modules top 30 most connected hub genes

**Fig 6:**
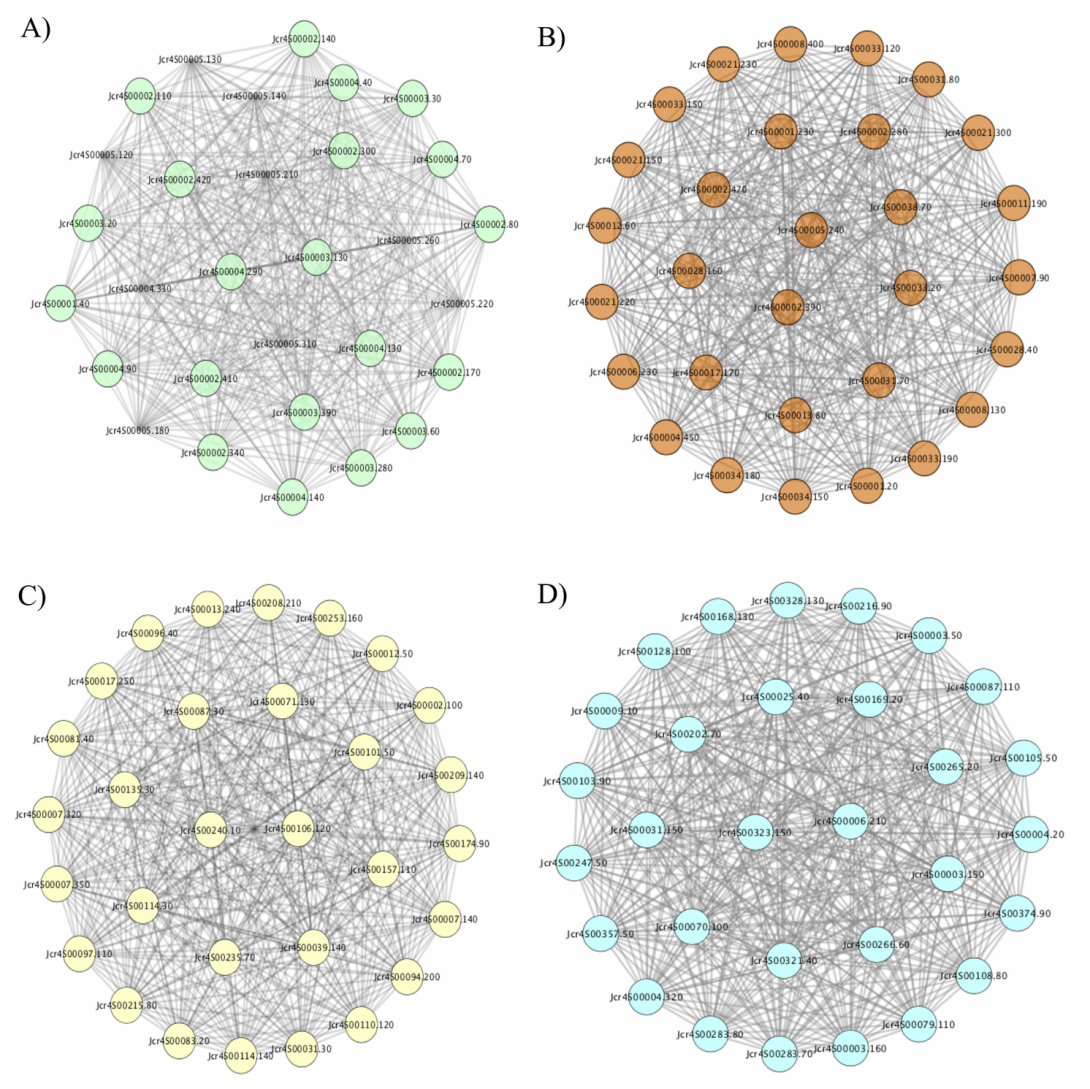
**The top-30 most connected hub genes extracted from the green4 (A), brown2 (B), antiquewhite2 (C) and lightskyblue4 (D) modules obtained from a weighted gene co-expression network analysis of *Jatropha curcas* inflorescence transcriptome.** The networks are drawn on Cytoscape with spring-embedded layout. Each node represent a gene and grey lines are the connecting edges.

We picked modules (green4, brown2, lightskyblue4 and antiquewhite2) corresponding to yield factors which are significant at p<0.01 for the dentification of hub genes. The genes present in each module was prioritized according to their intra-modular connectivity (the sum of connection strengths with other genes within the network). Genes with a high degree of connectivity were classified as hub genes. For each module, the top-30 most connected nodes were selected for functional classification of their annotated genes. Each module drawn with its top 30 most connected nodes is connected by 28–29 of directed edges and with a clustering coefficient ranging from 0.998–1. The shortest paths for each module is 870 (100%) and the connected component is one (Fig 6).

Table 2 shows the distribution of functionally annotated hub genes present in brown2, green4, lightskyblue4 and antiquewhite2 modules, from which genes were automatically and manually assigned. The hub genes are classified into 9 different groups: receptor and protein kinase, hypothetical/ uncharacterized and unknown protein, ROS generation and scavenging, transcription and translation, transcription factors, transport, chloroplast, mitochondria and cell differentiation and formation. For each module, about 5–9 genes encoding receptor and protein kinases are observed and indicated in parentheses: brown2 module (8), green4 (6), lightskyblue4 (5) and antiquewhite2 (9). Overall, 10 hypothetical/uncharacterized/unknown proteins are found in all modules. Only the lightskyblue4 and antiquewhite2 modules are found in transport process with 4 and 2 genes, respectively. The activity of chloroplast metabolism is highest in the lightskyblue4 module with 4 genes followed by both green4 and antiquewhite2 modules, with 3 genes each. The brown2 module with one gene showed the least activity. In contrast, the mitochondria metabolism shows the highest activity in green4 module, with 4 genes followed by lightskyblue4 (2 genes) and brown2 (1 gene) modules. No genes corresponding to mitochondria metabolism is found in the antiquewhite2 module. The antiquewhite2 module shows highest activity in cell differentiation/formation. All four modules participated in cell differentiation and formation: antiquewhite2 and lightskyblue4 modules with 6 genes each and green4 and brown2 modules with 3 genes in each. For transcription and translation, the green4 module shows the highest activity with 6 genes followed by the brown2 module with 4 genes, and lightskyblue4 and antiquewhite2 modules with 3 and 2 genes, respectively. The antiquewhite4 and green4 modules with 4 genes each show the highest activity in ROS scavenging and generation among the four modules, whereas both the lightskyblue4 and brown2 modules contain 2 genes each (Table 2).

**Table 2:** Functional classification of *Jatropha curcas* (inflorescence) annotated hub genes (top 30 most highly connected genes) present in the brown2, green4, lightskyblue4 and antiquewhite2 modules obtained from a weighted gene co-expression network analysis (WGCNA).

GeneID	Annotation	Module Colour
**Receptor and protein kinases**		
Jcr4S00001.20	IWS1 homolog A isoform X2	Brown2
Jcr4S00001.230	WD repeat-containing 91 homolog	Brown2
Jcr4S00002.280	tetratricopeptide repeat SKI3	Brown2
Jcr4S00005.240	mitogen-activated kinase kinase 2 isoform X1	Brown2
Jcr4S00007.90	BTB POZ and MATH domain-containing 2-like	Brown2
Jcr4S00008.400	plant intracellular Ras-group-related LRR 3	Brown2
Jcr4S00021.150	ras-related Rab7-like	Brown2
Jcr4S00033.190	Acyl- N-acyltransferase with RING FYVE PHD-type zinc finger	Brown2
Jcr4S00034.180	phosphatase 2C and cyclic nucleotide-binding kinase domain-containing isoform X1	Brown2
Jcr4S00002.340	Set domain isoform 1	Green4
Jcr4S00004.130	phospholipase D delta-like	Green4
Jcr4S00005.220	Serine arginine repetitive matrix 2 isoform 2	Green4
Jcr4S00005.130	zinc finger CCCH domain-containing 65 isoform X1	Green4
Jcr4S00005.210	AGC isoform 1	Green4
Jcr4S00003.150	zinc finger CCCH domain-containing 55-like	Lightskyblue4
Jcr4S00070.100	probable inactive receptor kinase At1g27190	Lightskyblue4
Jcr4S00202.70	LYR motif-containing 7 isoform 1	Lightskyblue4
Jcr4S00247.50	homeobox-leucine zipper ATHB-8	Lightskyblue4
Jcr4S00321.40	cyclin-H1-1 isoform X2	Lightskyblue4
Jcr4S00002.100	cyclin-dependent kinase B2-2	Antiquewhite2
Jcr4S00013.240	wall-associated receptor kinase-like 14	Antiquewhite2
Jcr4S00071.130	P-loop containing nucleoside triphosphate hydrolases superfamily isoform 1	Antiquewhite2
Jcr4S00094.200	F-box kelch-repeat At1g74510	Antiquewhite2
Jcr4S00097.110	signal peptide peptidase-like 2	Antiquewhite2
Jcr4S00106.120	tubby-like F-box 3	Antiquewhite2
Jcr4S00114.30	LIM domain-containing WLIM1-like	Antiquewhite2
Jcr4S00174.90	Regulatory NPR5	Antiquewhite2
Jcr4S00209.140	RPM1 interacting isoform 1	Antiquewhite2
Jcr4S00235.70	gibberellin receptor GID1C	Antiquewhite2
Jcr4S00004.40	calcium-dependent kinase 29	Green4
**Hypothetical/uncharacterized protein and unknown**		
Jcr4S00021.220	PREDICTED: uncharacterized protein LOC105637730	Brown2
Jcr4S00031.70	PREDICTED: uncharacterized protein LOC105632622	Brown2
Jcr4S00033.120	Unknown	Brown2
Jcr4S00034.150	ninja-family mc410	Brown2
Jcr4S00002.300	PREDICTED: uncharacterized protein LOC105635142 isoform X2	Green4
Jcr4S00003.130	PREDICTED: uncharacterized protein LOC105648311 isoform X2	Green4
Jcr4S00003.50	TITAN9 family	Lightskyblue4
Jcr4S00101.50	AF428345_1 AT3g54190 F24B22_150	Antiquewhite2
Jcr4S00114.140	vegetative incompatibility HET-E-1	Antiquewhite2
Jcr4S00208.210	UPF0481 At3g02645	Antiquewhite2
**Transcription factors**		
Jcr4S00012.60	CCAAT-displacement alternatively spliced product isoform 1	Brown2
Jcr4S00013.80	BEL1-like homeodomain 4	Brown2
Jcr4S00017.170	Sequence-specific DNA binding transcription factors	Brown2
Jcr4S00028.40	heat stress transcription factor B-2a	Brown2
Jcr4S00003.390	nuclear transcription factor Y subunit A-1	Green4
**Transport**		
Jcr4S00006.210	vacuolar sorting-associated 35B	Lightskyblue4
Jcr4S00087.110	vacuolar-processing enzyme	Lightskyblue4
Jcr4S00103.90	Major facilitator superfamily isoform 1	Lightskyblue4
Jcr4S00108.80	metal-nicotianamine transporter YSL1	Lightskyblue4
Jcr4S00110.120	vesicle transport v-SNARE 12	Antiquewhite2
Jcr4S00240.10	monosaccharide-sensing 2	Antiquewhite2
**Chloroplast metabolism**		
Jcr4S00008.130	chloroplast sensor chloroplastic isoform X1	Brown2
Jcr4S00003.280	RETICULATA-RELATED chloroplastic	Green4
Jcr4S00004.90	thioredoxin chloroplastic	Green4
Jcr4S00004.140	Plastocyanin family	Green4
Jcr4S00004.320	pentatricopeptide repeat-containing chloroplastic	Lightskyblue4
Jcr4S00128.100	chorismate mutase chloroplastic	Lightskyblue4
Jcr4S00266.60	ultraviolet-B receptor UVR8	Lightskyblue4
Jcr4S00283.80	chloroplast processing peptidase	Lightskyblue4
Jcr4S00007.350	phosphate chloroplastic	Antiquewhite2
Jcr4S00012.50	peptidyl-tRNA chloroplastic	Antiquewhite2
Jcr4S00017.250	pentatricopeptide repeat-containing chloroplastic	Antiquewhite2
**Mitochondria metabolism**		
Jcr4S00021.300	aceous RNase P chloroplastic mitochondrial-like	Brown2
Jcr4S00001.40	mitochondrial carrier MTM1	Green4
Jcr4S00002.410	FAD NAD(P)-binding oxidoreductase family	Green4
Jcr4S00004.290	probable mitochondrial intermediate mitochondrial	Green4
Jcr4S00005.140	37S ribosomal mitochondrial	Green4
Jcr4S00031.150	glutamate dehydrogenase 1	Lightskyblue4
Jcr4S00079.110	mitochondrial isoform X1	Lightskyblue4
**Cell differentiation/formation**		
Jcr4S00021.230	CONTINUOUS VASCULAR RING 1	Brown2
Jcr4S00031.80	arabinogalactan peptide 13-like	Brown2
Jcr4S00033.20	Spindle assembly abnormal 6	Brown2
Jcr4S00002.110	FAR1-RELATED SEQUENCE 5-like	Green4
Jcr4S00002.420	QWRF motif-containing 8	Green4
Jcr4S00004.70	probable prolyl 4-hydroxylase 3	Green4
Jcr4S00169.20	peptidyl-prolyl cis-trans isomerase CYP65 isoform X1	Lightskyblue4
Jcr4S00216.90	probable xyloglucan endotransglucosylase hydrolase 28	Lightskyblue4
Jcr4S00265.20	glucan endo-1 family	Lightskyblue4
Jcr4S00283.70	DPP6 N-terminal domain	Lightskyblue4
Jcr4S00328.130	fasciclin-like arabinogalactan 1	Lightskyblue4
Jcr4S00357.50	endoglucanase 2-like	Lightskyblue4
Jcr4S00007.140	AWPM-19-like membrane family	Antiquewhite2
Jcr4S00031.30	tubulin alpha-3 chain	Antiquewhite2
Jcr4S00083.20	pollen-specific leucine-rich repeat extensin 2	Antiquewhite2
Jcr4S00087.30	EARLY FLOWERING 3	Antiquewhite2
Jcr4S00135.30	GRF1-interacting factor 1-like	Antiquewhite2
Jcr4S00215.80	callose synthase 1	Antiquewhite2
**ROS generation and scavenging**		
Jcr4S00002.390	chaperone dnaJ 10-like	Brown2
Jcr4S00004.450	BAG family molecular chaperone regulator 7	Brown2
Jcr4S00003.30	DNA binding,zinc ion binding,DNA isoform 3	Green4
Jcr4S00003.60	ubiquitin-conjugating enzyme E2 20	Green4
Jcr4S00005.180	U-box domain-containing 34	Green4
Jcr4S00005.260	UBP1-associated s 1C	Green4
Jcr4S00004.20	E3 ubiquitin- ligase ATL23	Lightskyblue4
Jcr4S00009.10	U-box domain-containing 14	Lightskyblue4
Jcr4S00168.130	lipopolysaccharide-induced tumor necrosis factor-alpha factor homolog	Antiquewhite2
Jcr4S00007.320	peptide methionine sulfoxide reductase B5-like	Antiquewhite2
Jcr4S00039.140	PAT1 homolog 1-like isoform X1	Antiquewhite2
Jcr4S00081.40	FIZZY-RELATED 3	Antiquewhite2
**Transcription and translation**		
Jcr4S00002.470	eukaryotic translation initiation factor isoform 4G-1	Brown2
Jcr4S00006.230	snRNA-activating complex subunit 4	Brown2
Jcr4S00011.190	endoribonuclease Dicer homolog 1	Brown2
Jcr4S00028.160	probable transcriptional regulator SLK2	Brown2
Jcr4S00033.150	suppressor of gene silencing 3	Brown2
Jcr4S00002.80	DEAD-box ATP-dependent RNA helicase 42	Green4
Jcr4S00002.140	splicing factor U2af small subunit B	Green4
Jcr4S00002.170	U5 small nuclear ribonucleo 200 kDa helicase-like	Green4
Jcr4S00003.20	nuclear cap-binding subunit 1	Green4
Jcr4S00005.120	5—nucleotidase	Green4
Jcr4S00025.40	family transposase isoform 1	Lightskyblue4
Jcr4S00105.50	telomerase Cajal body 1	Lightskyblue4
Jcr4S00323.150	deoxyhypusine synthase	Lightskyblue4
Jcr4S00096.40	high mobility group B 6	Antiquewhite2
Jcr4S00157.110	Nuclear factor related to kappa-B-binding	Antiquewhite2

The geneIDs listed are the same as that deposited in NCBI repository. The module colour with putative function were generated from WGCNA of the transcriptome, yield (number of flowers per inflorescence, number of seeds per plant, total weight of seeds per plant and number of fruits per plant) and simple soluble sugar (glucose and fructose) data of *J*. *curcas* inflorescence: green4 module corresponds to number of seeds per plant, total weight of seeds per plant and number of fruits per plant, antiquewhite2 module corresponds to number of flowers per inflorescence and both brown2 and lightskyblue4 modules correspond to fructose and glucose.

### Antiquewhite2 and green4 modules: Hub genes for gibberellin (GA) signaling and flowering

The phytohormone, gibberellins (GAs) are tetracyclic diterpenoids that affect multiple aspect of plant growth and development processes; leaf expansion, stem/internode and root elongation, floral induction, flower development, fruit set and development/growth, and seed germination [53, 54]. Studies on GAs have made tremendous discoveries especially in regard to plant reproduction. As such, the role of GA is evident in the vegetative to reproductive state transitions, formation of inflorescence meristems and subsequent floral meristems, growth of floral organs and development of microspores and megagametophytes (reproductive cells). The GA biosynthesis genes such as the GA20-oxidases (GA20ox) and GA3ox have been up-regulated upon pollination and ovule fertilization [55] and others have shown positive effect of GA treatment on ovule fertilization and subsequent fruit initiation [56].

Over the years, numerous studies have been conducted on exogenous treatment of GA on *J*. *curcas* plants across the globe. In *J*. *curcas*, the exogenous application of gibberellic acid (GA_3_) had adversely impacted the physiology of the plant shoot system. In 2-year-old plants, a high expression of putative branching regulator genes, *BRANCHED1* and *BRANCHED2* in GA_3_ treated plants suggest that gibberellin is a positive regulator of *J*. *curcas* shoot branching. In addition, apparent number of lateral branchings were observed in the GA_3_ treated plants [57]. In another study conducted on 4-year-old plants in China, the exogenous application of GA_3_ inhibited floral initiation and subsequent flowering [58]. However, in contrast to these findings, similar studies performed in China have indicated that flowering characteristics such as total flower number, female-to-male flower ratio and floral bud differentiation were improved upon GA_3_ treatment on the inflorescence [59, 60].

The antiquewhite2 module identified in this study contains hub genes for flowers/inflorescence. These genes could potentially take part in GA signaling primarily due to the presence of a GA receptor gene, the *GIBBERELLIN INSENSITIVE DWARF1 (GID1C)* and other genes related to flowering (*pollen-specific leucine-rich repeat extensin 2*, *EARLY FLOWERING 3*, *FIZZY-RELATED 3*, *pentatricopeptide repeat-containing chloroplastic*), and, cell elongation and division (*cyclin-dependent kinase B2-2* and *wall-associated receptor kinase-like 14*). The GA pathways are extensively described in Arabidopsis and rice. In Arabidopsis, gibberellin perception is controlled by three types of GA receptors, GID1A, GID1B and GID1C [61, 62]. Gibberellin binds to one of these three receptors to form the GID1-GA complex. Following a conformational change in the N-terminus region of GA, the GID1-GA binds to a GA-signaling repressor DELLA protein. DELLAs are growth-inhibitory proteins and therefore, the disruption will promote mitotic cycle for cell proliferation and division required for plant reproductive growth. The GID1-GA-DELLA complex interacts with complementary F-box SLEEPY1 (SLY1)/GID2 to cause polyubiquitination and subsequent degradation of the DELLA proteins [61, 62, 63]. In antiquewhite2 module, the presence of both the *tubby-like F-box 3* and *F-box kelch-repeat At1g74510* represent the F-box proteins, also a class of the ubiquitin protein ligases called the E3 ligases. Under the ubiquitin-proteasome pathways, cellular proteins are targeted to degradation by the 26S proteasome during GA signaling. Dendrogram of module eigengenes (MEs) indicates strong correlations between MEantiquewhite2 and MEgreen4 (Fig 3). In parallel with the role of antiquewhite2 module in GA signaling, the presence of *ubiquitin-conjugating enzyme E2 20*, *U-box domain-containing 34* and *UBP1-associated s 1C* genes in green4 module of *J*. *curcas* inflorescence may further corroborate with the ubiquitination event in GA signaling pathway (Table 1). The green4 module correspond to flowers/inflorescence, fruits/plant, seeds/plant and seed weight (g)/plant and the role of GA signaling on yield-related traits has been described in *J*. *curcas* [59, 60]. The correlation between the antiquewhite2 and green4 modules may suggest an occurrence of GA signaling under the control of both modules for reproductive-related responses such as flowering, fruiting and subsequent seed yield.

### Brown2 and lightskyblue4: Hub genes for sugar signaling

Simple sugars (glucose, fructose and sucrose) produced by the photosynthetic autotrophs are essential structural components and energy units. In addition, they are used as metabolic messengers for the co-ordination of metabolic activities between tissues in a plant body. These sugars are able to regulate photosynthetic activity by controlling the modulation of gene expression and enzyme activities involved in the photosynthesis metabolism, in addition to others such as the carbohydrate and nitrogen metabolism, signal transduction and stress responses in plants [19, 42, 64, 65]. Sugar sensing and signaling controls growth and development of plants throughout their entire life cycle and the process has been especially critical in plant reproduction. During floral transition and flowering, mobilization of starch and an increase of carbohydrate export to the leaf have been shown in Arabidopsis [66]. Both brown2 and lightskyblue4 modules correspond to glucose and fructose concentration in *J*. *curcas* inflorescence. In plants, protein phosphorylation and dephosphorylation is among the most common mechanism employed for signal transduction. In glucose sensing and regulatory effect, transport and phosphorylation of sugar is an integral part of the mechanism. Glucose transport analogues are either transported or phosphorylated with the presence of a membrane receptor, generally the sugar kinase homologue [18, 19]. A variety of protein kinases have been described in sugar signaling and regulatory effect. The brown2 module contain the highest number of protein kinases and both *phosphatase 2C and cyclic nucleotide-binding kinase domain-containing isoform X1* and *mitogen-activated kinase kinase 2 isoform X* genes may have been involved in glucose signaling for the regulation of reproduction process in *J*. *curcas* inflorescence. Likewise, the *probable inactive receptor kinase At1g27190* gene in the lightskyblue module may have played a similar role.

Arabinogalactan proteins (AGPs) is a big family of hydroxyproline-rich glycoproteins, mainly found adhered on plasma membrane by a glycosylphosphatidylinositol (GPI) anchor [67, 68]. Composed of protein cores surrounded by carbohydrates, they play an important role as signaling molecules in plants [69]. We identified 2 genes in modules corresponding to glucose and fructose; lightskyblue module contain the *fasciclin-like arabinogalactan 1* gene and the brown2 module contain the *arabinogalactan peptide 13-like gene* (Table 1). Transport activity was fairly high in the lightskyblue4 module. The presence of *vacuolar sorting-associated 35B*, *vacuolar-processing enzyme*, *Major facilitator superfamily isoform 1* and *metal-nicotianamine transporter YSL1* genes may suggest sugar transport at membrane, a process observed during sugar signaling response [18].

### Breeding opportunities

The WGCNA analysis clearly demonstrated that the yield-related phenotypes of *J*. *curcas* are tightly regulated by gene networks. Possibly, the hub genes for physical traits and simple soluble sugars are interconnected, as indicated by the dendrogram of module eigengenes. Our stringent count-data filtration cut-off, set at cpm value of <5 compromises the exclusion of a validation experiment. Based on functional gene analyses available in literature, the list of candidate players presented here are fundamentally potent for the enhancement of *J*. *curcas* reproductive traits and overall plant yield performance. Quantitative traits improvements are particularly challenging because these traits are controlled by a large number of genes/loci with each gene contributing a relatively small effect. Presently, genetic engineering approaches are widely available and these tools have been adopted for *J*. *curcas* improvements [70]. Nevertheless, the production of an outstanding *J*. *curcas* planting material with superior performance remain hampered. The RNA interference or the RNAi silencing mechanism has emerged as a powerful tool for *in planta* gene expression manipulation and have been actively adopted in *J*. *curcas* [71,72,73,74]. The mechanism silences the expression of a target gene, introduced through its silencing machinery [75]. In this study, the candidate genes identified and their putative involvement in the described mechanisms may require either an RNAi based functional analyses or a mutant analysis prior to downstream application in *J*. *curcas* breeding programs.

## Conclusions

The co-expression network constructed using the RNA-sequencing based expression profiles predicted the candidate genes for modules corresponding to *J*. *curcas* yield factors (simple soluble sugars and plant physical traits). Simple soluble sugar analysis in *J*. *curcas* inflorescence revealed a relative ratio of *glc*: *fru*: *suc* at 1.8:1.5:1 and both the brown2 and lightskyblue4 modules were found corresponding to glucose and fructose only. Modules corresponding to sucrose were not obtained. Modules corresponding to physical data were described as following; green4 for flowers/inflorescence, fruits /plant, seed weight and seed number, and antiquewhite2 for flowers/ inflorescence only. The top 30 most-connected genes established for yield-related modules (brown2, lightskyblue4, green4, antiquewhite2) will significantly ease the selection of candidate genes targeted for *J*. *curcas* reproductive biology improvement. Our findings provide an essential list of predicted candidate genes and is likely to inform further research in an attempt to improve *J*. *curcas* yield factors.

## Supporting information

S1 Fig Quantification of glucose (*glc*), fructose (*fru*) and sucrose (*suc*) in 2-year-old *Jatropha curcas* inflorescence (UKM JC-17 accession). A) HPLC-ELSD chromatograms of simple soluble sugars; A (i), standard peaks and A (ii), sample peaks are indicated at the following retention times (rt): *fru* at 8.6, *glc* at 10.8 and *suc* at 16.0. The y-axis represents peak area expressed as mass absorbance unit (mAU) and the x-axis represents the rt in minutes. B) Standard curves of simple soluble sugars at 2.5–10 mM: B (i), *fru*; B (ii) and *glc*; B (iii), *suc*.
(PDF)

S2 Fig Network topology analysis of *Jatropha curcas* inflorescence transcriptome data for a range of soft-thresholding powers (1–20). The left panel indicates the scale-free fit index (y-axis) as a function of soft-thresholding power (x-axis). Red line represents a first time plateau point. The right panel shows mean connectivity (y-axis) as a function of soft-thresholding power (x-axis).
(PDF)
