# Supplementary material for: An integration of phenotypic and transcriptomic data analysis reveals yield-related hub genes in Jatropha curcas inflorescence
Source: PLoS One. 2018 Sep 21;13(9):e0203441. doi: 10.1371/journal.pone.0203441 (PMC6150480; doi:10.1371/journal.pone.0203441)
Supplement: S1 Fig — A) HPLC-ELSD chromatograms of simple soluble sugars; A (i), standard peaks and A (ii), sample peaks are indicated at the following retention times (rt): fru at 8.6, glc at 10.8 and suc at 16.0. The y-axis represents peak area expressed as mass absorbance unit (mAU) and the x-axis represents the rt in minutes. B) Standard curves of simple soluble sugars at 2.5–10 mM: B (i), fru; B (ii) and glc; B (iii), suc. (PDF) [file pone.0203441.s001.pdf]

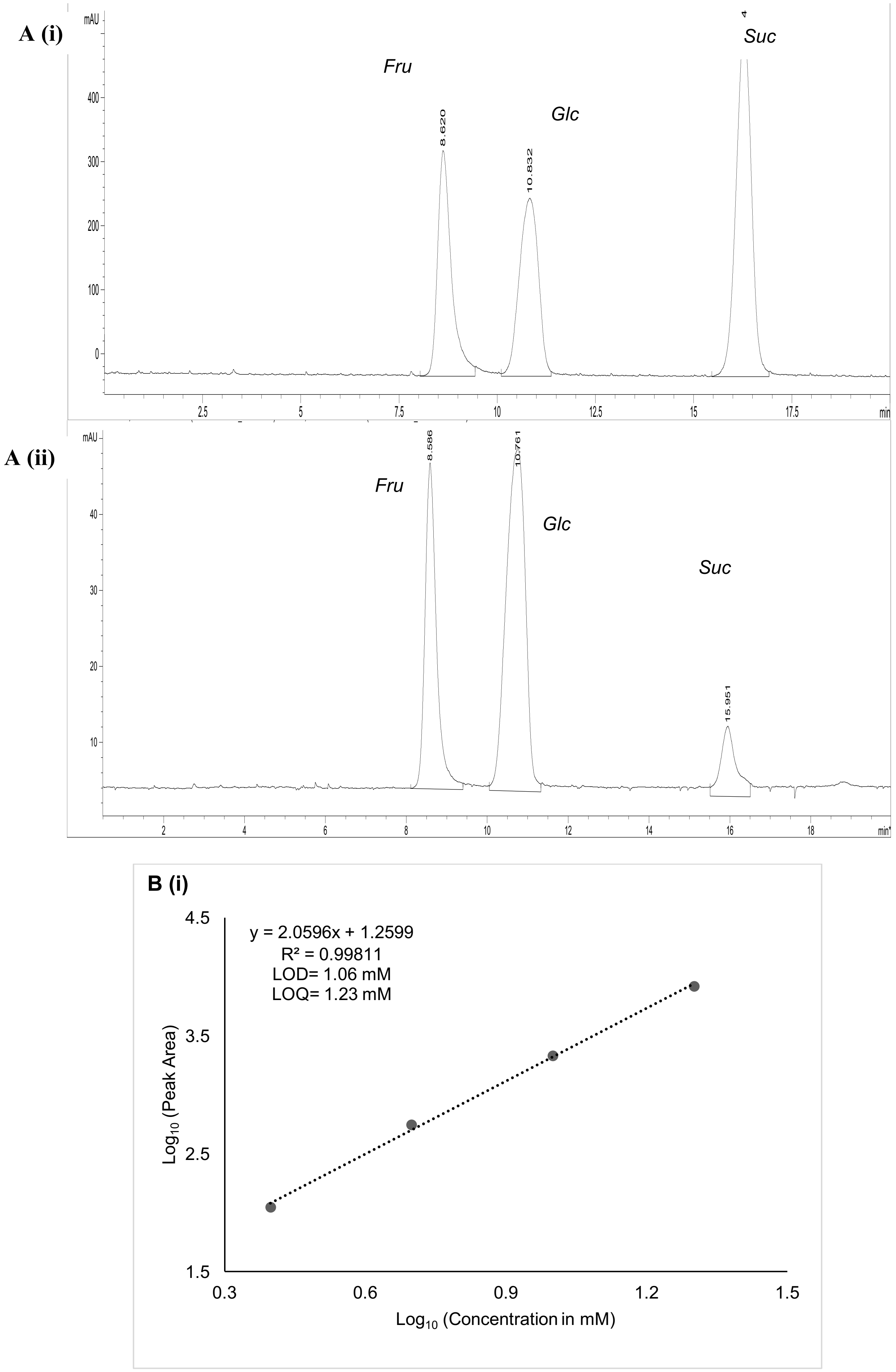

A (i)
mAU
Fru
Glc
Suc
8.620
10.832
400
300
200
100
0
2.5
5
7.5
10
12.5
15
17.5
min
A (ii)
mAU
Fru
Glc
Suc
8.586
10.761
15.951
40
30
20
10
2
4
6
8
10
12
14
16
18
min
B (i)
y = 2.0596x + 1.2599
R² = 0.99811
LOD= 1.06 mM
LOQ= 1.23 mM
4.5
3.5
2.5
1.5
0.3
0.6
0.9
1.2
1.5
Log10 (Peak Area)
Log10 (Concentration in mM)

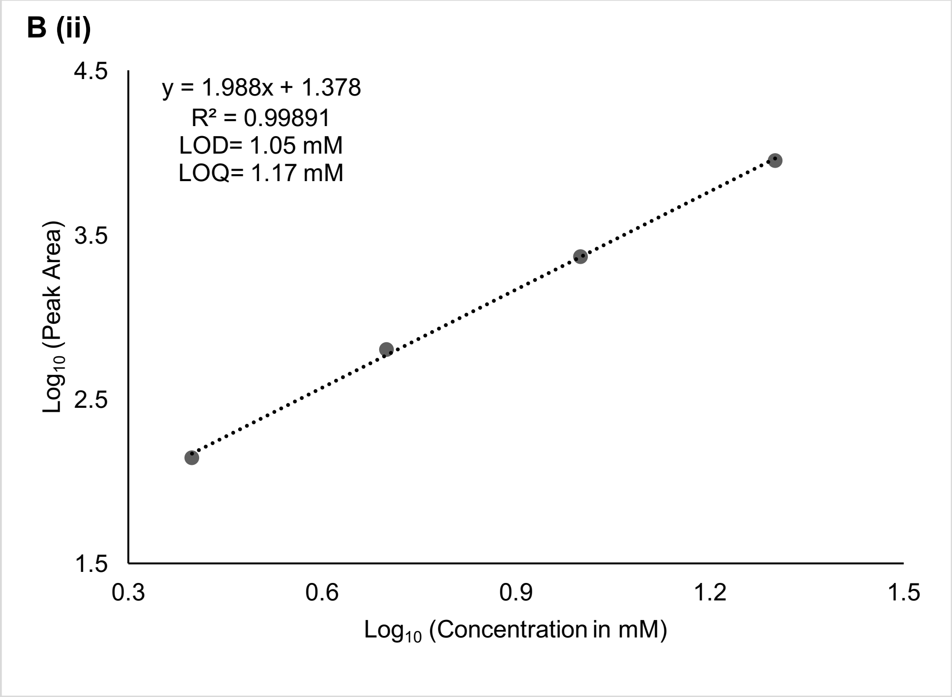

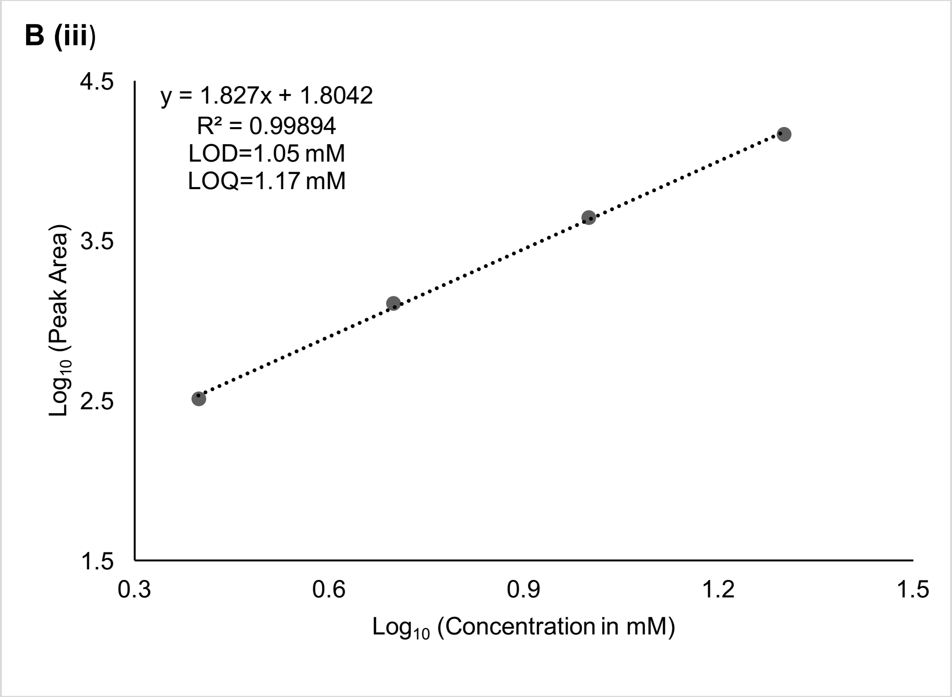

S1 Fig. Quantification of glucose (*glc*), fructose (*fru*) and sucrose (*suc*) in 2-year-old *Jatropha curcas* inflorescence (UKM JC-17 accession). A) HPLC-ELSD chromatograms of simple soluble sugars; A (i), standard peaks and A (ii), sample peaks are indicated at the following retention times (rt): *fru* at 8.6, *glc* at 10.8 and *suc* at 16.0. The y-axis represents peak area expressed as mass absorbance unit (mAU) and the x-axis represents the rt in minutes. B) Standard curves of simple soluble sugars at 2.5-10 mM: B (i), *fru*; B (ii) and *glc*; B (iii), *suc*.
