# Supplementary material for: An integration of phenotypic and transcriptomic data analysis reveals yield-related hub genes in Jatropha curcas inflorescence
Source: PLoS One. 2018 Sep 21;13(9):e0203441. doi: 10.1371/journal.pone.0203441 (PMC6150480; doi:10.1371/journal.pone.0203441)
Supplement: S2 Fig — The left panel indicates the scale-free fit index (y-axis) as a function of soft-thresholding power (x-axis). Red line represents a first time plateau point. The right panel shows mean connectivity (y-axis) as a function of soft-thresholding power (x-axis). (PDF) [file pone.0203441.s002.pdf]

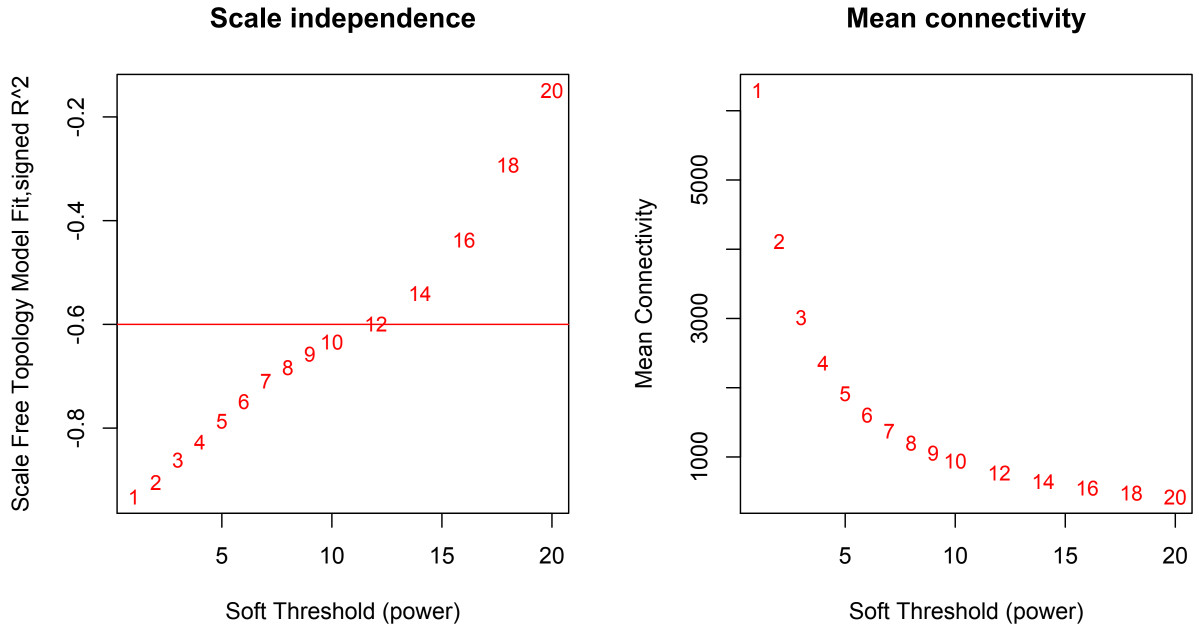

S2 Fig. Network topology analysis of *Jatropha curcas* inflorescence transcriptome data for a range of soft-thresholding powers (1-20). The left panel indicates the scale-free fit index (y-axis) as a function of soft-thresholding power (x-axis). Red line represents a first time plateau point. The right panel shows mean connectivity (y-axis) as a function of soft-thresholding power (x-axis).
